# Abnormal Functional Connectivity of Hippocampal Subdivisions in Obstructive Sleep Apnea: A Resting-State Functional Magnetic Resonance Imaging Study

**DOI:** 10.3389/fnins.2022.850940

**Published:** 2022-04-25

**Authors:** Xiang Liu, Liting Chen, Wenfeng Duan, Haijun Li, Linghong Kong, Yongqiang Shu, Panmei Li, Kunyao Li, Wei Xie, Yaping Zeng, Dechang Peng

**Affiliations:** ^1^Department of Radiology, The First Affiliated Hospital of Nanchang University, Nanchang, China; ^2^Medical Imaging Center, First Affiliated Hospital of Jinan University, Guangzhou, China

**Keywords:** obstructive sleep apnoea, hippocampus, cognitive, impairment, fMRI, functional connectivity

## Abstract

The hippocampus is involved in various cognitive function, including memory. Hippocampal structural and functional abnormalities have been observed in patients with obstructive sleep apnoea (OSA), but the functional connectivity (FC) patterns among hippocampal subdivisions in OSA patients remain unclear. The purpose of this study was to investigate the changes in FC between hippocampal subdivisions and their relationship with neurocognitive function in male patients with OSA. Resting-state fMRI were obtained from 46 male patients with untreated severe OSA and 46 male good sleepers. The hippocampus was divided into anterior, middle, and posterior parts, and the differences in FC between hippocampal subdivisions and other brain regions were determined. Correlation analysis was used to explore the relationships between abnormal FC of hippocampal subdivisions and clinical characteristics in patients with OSA. Our results revealed increased FC in the OSA group between the left anterior hippocampus and left middle temporal gyrus; between the left middle hippocampus and the left inferior frontal gyrus, right anterior central gyrus, and left anterior central gyrus; between the left posterior hippocampus and right middle frontal gyrus; between the right middle hippocampus and left inferior frontal gyrus; and between the right posterior hippocampus and left middle frontal gyrus. These FC abnormalities predominantly manifested in the sensorimotor network, fronto-parietal network, and semantic/default mode network, which are closely related to the neurocognitive impairment observed in OSA patients. This study advances our understanding of the potential pathophysiological mechanism of neurocognitive dysfunction in OSA.

## Introduction

Obstructive sleep apnoea (OSA) is one of the most common sleep disorders. It is characterized by the repeated collapse of the upper respiratory tract during sleep, resulting in occlusion or stenosis, sleep fragmentation, and a decrease in blood oxygen saturation. Among adults, approximately 14% of men and 5% of women are diagnosed with OSA. Obesity and increasing age are associated with an increased incidence of OSA (Patel, 2019; Wang et al., 2020). Chronic intermittent hypoxia and abnormal sleep rhythms lead to decreased quality of life and long-term health problems, including complications such as high blood pressure, cardiovascular damage, stroke, depression, chronic kidney disease, and insomnia (Kerner and Roose, 2016; Yayan et al., 2017). In addition, OSA presents with a variety of neurocognitive impairments, including reductions in attention, alertness, executive function, long-term visual and verbal memory, cognitive function, and visuospatial and visual construction (Lal et al., 2012; Bucks et al., 2013; Delhikar et al., 2019; Bubu et al., 2020). Although earlier studies on OSA have proposed several mechanisms of impairment, including brain tissue damage caused by intermittent hypoxia or ischemia, and emotional and cognition-related network dysfunction caused by sleep disturbance and fragmented sleep (Goldstein and Walker, 2014; Rosenzweig et al., 2015), the exact neural mechanisms of cognitive impairment in OSA patients remain unclear.

The hippocampi of patients with OSA exhibit damage, which may contribute to the disordered memory, autonomic nervous system dysfunction, and depressive symptoms. Previous findings indicate that decreased hippocampal volume (Weng et al., 2014; Owen et al., 2021) and abnormal functional connectivity (FC) between the hippocampus and the whole brain (Zhou et al., 2020a) are associated with memory impairment in patients with OSA, and levels of metabolites that suggest inflammation and glial activation have also been observed (Sarma et al., 2014). Song et al. (2018) reported that the disruption of functional network patterns between the hippocampus and the thalamus, superior temporal gyrus, insula, posterior cingulate cortex, and other structures is the basis for the emotional regulation defects of depression and anxiety. Chang et al. (2020) observed that the apnoea-hypopnea index (AHI) was associated with neural activity in the hippocampal region associated with the default mode network, and decreased cerebrovascular reactivity was the pathological mechanism of decreased hippocampal neural activity. These studies have focused on exploring structural or functional changes in the hippocampus as a whole, but the specific areas of damage are unclear. Therefore, the structural and functional changes occurring in the hippocampal subregions have been gaining more attention.

Understanding the functional and structural impairments in the hippocampal subregions will provide insight into the neurocognitive mechanisms associated with OSA. Early rodent electrophysiological and recent gene expression studies showed that the dorsal and ventral parts of the hippocampus have separable response characteristics (Thompson et al., 2008; Kheirbek and Hen, 2011; Lee et al., 2017; Floriou-Servou et al., 2018). These studies have contributed to our understanding of the functional specialization pattern of the hippocampal long axis, which provides an anatomical and genetic basis for neuroimaging research to explore the hippocampal subregion. Seed region-of-interest based FC analysis (Zarei et al., 2013), structural magnetic resonance imaging (MRI) (Pipitone et al., 2014), and diffusion tensor imaging (Treit et al., 2018) revealed that the hippocampus is functionally and structurally divided into different subregions. Qin et al. (2016) demonstrated that enhanced FC between the posterior hippocampus and the widely distributed cortical regions was related to sensory and reward processes. Meanwhile, graph theory analysis indicated that the centrality of anterior hippocampus nodes was low, which confirmed the heterogeneous FC pattern of the anterior and posterior hippocampus. Dugre et al. (2021) confirmed the FC patterns of different hippocampal subregions during episodic memory tasks in patients with schizophrenia, finding that the connectivity defects between the posterior hippocampus and default mode network region were related to the encoding retrieval flip mode and the increased connectivity between the posterior hippocampus and the intracalcarine cortex was closely related to memory accuracy. However, the FC pattern of the hippocampal subregions in patients with OSA remains unclear.

Therefore, we hypothesized that FC abnormalities between the anterior and posterior axes of the hippocampus that participate in cognitive functions such as memory would be found in patients with OSA. Thus, we aimed to explore the FC between regions of interest (ROIs) in the anterior, middle, and posterior subdivisions of the hippocampus and the whole brain to assess the changes in FC patterns in patients with OSA. We also explored the relationships between the abnormal FC in these hippocampal subdivisions and clinical characteristics in patients with OSA.

## Materials and Methods

### Subjects

In this study, 46 untreated male patients with severe OSA diagnosed by polysomnography and an AHI of >30/h (OSA group) were recruited from the Department of Respiratory and Otorhinolaryngology of The First Affiliated Hospital of Nanchang University between September 2016 and June 2021. In addition, 46 good sleepers (GS group) of similar ages and education levels and with an AHI of <5/h were recruited from the community (Table 1). The inclusion criteria were based on the guidelines for the diagnosis of OSA syndrome issued by the American Sleep Medical Association in 2017 (Kapur et al., 2017). The exclusion criteria for all subjects were as follows: (1) suffering from other sleep-related diseases such as insomnia; (2) history of diabetes and cardiovascular diseases; (3) history of drug abuse, alcoholism, or psychotropic drug use; (4) neurodegenerative diseases, depression, brain trauma, epilepsy, or other neuropsychiatric diseases; (5) recent history of surgery, trauma, infection, or other systemic diseases; and (6) contraindications for MRI scanning (such as steel plate implantation). This study was approved by the Medical Ethics Committee of our hospital, and all subjects participated voluntarily and signed an informed consent form.

**TABLE 1:** Demographic and clinical data of obstructive sleep apnoea (OSA) and good sleepers (GS).

Category	OSA(*n* = 46)	GSs(*n* = 46)	*t*-Value	*P*-Value
Age (year)*^a^*	38.15 ± 9.64	38.32 ± 11.72	–0.078	0.938
BMI (Kg/m^2^)*^a^*	27.51 ± 3.29	23.08 ± 1.96	7.827	< 0.001
Education (year)*^b^*	12.10 ± 3.17	11.43 ± 3.50	0.966	0.337
AHI,/hour*^a^*	27.51 ± 3.29	23.08 ± 1.96	18.529	< 0.001
Total sleep time (min)*^b^*	372.2 ± 83.88	398.30 ± 18.93	–2.054	0.045
Sleep efficiency (%)*^b^*	0.84 ± 0.178	0.919 ± 0.057	–2.847	0.006
LSaO2 (%)*^a^*	66.40 ± 12.56	92.15 ± 5.80	–12.71	< 0.001
MSaO2 (%)*^a^*	90.62 ± 4.48	96.17 ± 2.38	–7.46	< 0.001
N1 stage (%)*^a^*	31.26 ± 17.37	10.21 ± 3.72	8.034	< 0.001
N2 stage (%)*^a^*	39.45 ± 14.65	47.86 ± 5.55	–3.641	0.001
N3 stage (%)*^b^*	21.96 ± 18.37	21.15 ± 4.53	0.293	0.771
REM (%)*^b^*	7.47 ± 8.12	21.89 ± 7.47	–8.858	< 0.001
SaO2 < 90%*^b^*	30.55 ± 20.71	0.269 ± 0.177	9.914	< 0.001
AI*^b^*	39.54 ± 23.63	11.93 ± 2.79	7.867	< 0.001
MoCA*^a^*	25.2 ± 2.08	27.73 ± 1.38	–6.824	< 0.001
ESS*^a^*	11.97 ± 3.83	3.39 ± 2.17	13.2	< 0.001

*GSs, normal sleep group; AHI, apnea hypopnea index; LSaO2, minimum blood oxygen saturation; MSaO2, average blood oxygen saturation; REM, rapid eye movement; SaO2 < 90%, percentage of total sleep time with oxygen saturation less than 90 AI, arousal index; ESS, Epworth sleepiness scale. ^a^Student’s t-test. ^b^Mann-Whitney U-test.*

### Clinical Scale Evaluation and Polysomnography

Daytime sleepiness was assessed using the Epworth Sleepiness Scale (ESS) and neurocognitive function was assessed using the Montreal Cognitive Assessment (MoCA). The MoCA includes visual space and execution, naming, memory, attention, language, abstract thinking, calculation, and orientation (Nasreddine et al., 2005). The highest possible MoCA score is 30 points, and scores of <26 indicate cognitive impairment. If the number of years of education was <12 years, one point was added to the total score to adjust for education. All subjects underwent ≥7 h of polysomnography at night and were asked to not drink coffee or alcohol and not to take sleeping pills on the same day. All of the above assessments were conducted in like manner by professionally trained doctors, and all examinations were carried out in the same order for all participants. Based on the guidelines of the American Sleep Medical Association, obstructive apnoea was defined as an airflow reduction of ≥90% persisting for >10 s and accompanied by continuous breathing effort; hypopnea was defined as an airflow reduction of 30–89% persisting for ≥10 s and accompanied by a decrease of ≥4% in oxygen saturation and/or electroencephalogram (EEG) awakening. The AHI was calculated as the average number of apnoea and hypopnea events experienced per hour during sleep. The arousal index was calculated as the average number of EEG arousal events per hour of sleep (Berry et al., 2012).

### MRI Data Acquisition

All images were captured on a 3.0 Tesla MRI system with an eight-channel phased-array head coil (Siemens, Munich, Germany). The subjects were instructed to lie quietly on the examination bed with their eyes closed, wore earplugs and goggles, had their head position stabilized with sponges, and were asked to try to clear their mind. First, conventional T1-weighted imaging was performed using the following parameters: repetition time (TR) = 4,000 ms, echo time (TE) = 113 ms, thickness = 5 mm, gap = 1.5 mm, field of view (FOV) = 220 mm × 220 mm, and slice thickness = 19. T2-weighted imaging was then performed using the following parameters: TR = 4,000 ms, TE = 113 ms, thickness = 5 mm, gap = 1.5 mm, FOV = 220 mm × 220 mm, and slice thickness = 19. Resting-state whole-brain blood oxygenation level dependent (BOLD) data were collected by echo-planar imaging using the following parameters: TR = 2,000 ms, TE = 30 ms, flip angle = 90°, FOV = 230 mm × 230 mm, matrix = 64 × 64, layer number = 30, slice thickness = 4.0 mm, interval = 1.2 mm, and scanning time of approximately 8 min. Finally, high-resolution three-dimensional T1-weighted images were obtained using the following brain volume sequence parameters: TR = 1,900 ms, TE = 2.26 ms, thickness = 1.0 mm, gap = 0.5 mm, FOV = 250 mm × 250 mm, matrix = 256 × 256, and flip angle = 9°, sagittal slices. After the MRI scan, all participants were asked if they had fallen asleep during the scan, and all images were examined by two senior radiologists to rule out significant brain damage.

### Data Preprocessing

First, the MRIcro^1^ software package was used to evaluate the fMRI data, and data with incomplete image range, head movement, or large artifacts were removed. Next, based on the MATLAB 2012A (Mathworks, Natick, MA, United States) platform and the SPM 8^2^ software package, the DPARSF V2.3 software package was used to pre-process the original data. The main steps were as follows: (1) The original DICOM format file was converted to NIFTI format. (2) The first ten time points were removed, and the remaining 230 volumes were corrected for time (all images acquired within the TR were corrected to the same time point) and head motion (removal of swallowing, hemodynamics, respiration, etc.) to eliminate physiological noise and potential head motion-related artifacts; these factors were compared between groups to assess their relative impact (Saad et al., 2012). (3) Data were excluded if during fMRI scanning the maximum shift in any direction exceeded 2.0 mm, the angle rotation along any axis exceeded 2.0°, or the frame displacement of any of the 230 volumes exceeded 2.5 standard deviations (Van Dijk et al., 2012). (4) Diffeomorphic Anatomical Registration Through Exponentiated Lie Algebra was used to convert images from an individual’s original space to the Montreal Neurological Institute (MNI) space, and the image was resampled to 3 mm^3^ × 3 mm^3^ × 3 mm^3^ voxels. (5) The image was then smoothed with a 6 mm half-width Gaussian smoothing kernel. (6) To further reduce confounding factors, nuisance covariates, including white matter signals, cerebrospinal fluid, whole-brain signals, and 24-parameter head movements were regressed from the time series of all voxels using linear regression (Zhu et al., 2021). (7) A temporal bandpass filter (0.01–0.08 Hz) was used to reduce low-frequency drift and physiological high-frequency noise.

### Definition of Regions of Interests and Functional Connectivity Calculations Across Hippocampal Subdivisions

**FIGURE 1:**
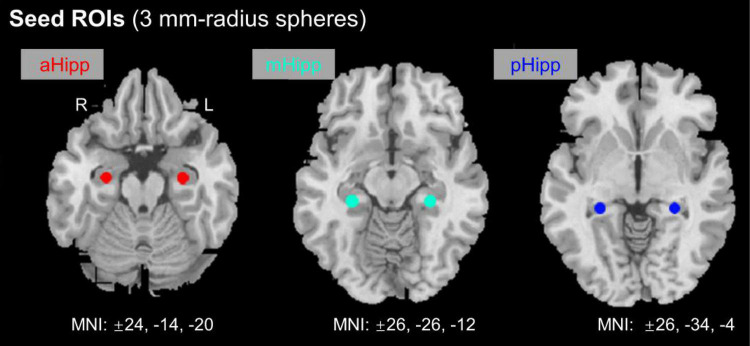
Location of nodes along the anterior and posterior axes of the hippocampus. aHipp, anterior hippocampus; mHipp, middle hippocampus; pHipp, posterior hippocampus.

Based on previous guidelines, the hippocampus was anatomically divided into anterior, middle, and posterior regions. In this study, six ROIs were defined along the longitudinal axis of the left and right hippocampus with a radius of 3 mm (Figure 1). Each side of the hippocampus had three ROIs: the head (anterior, aHipp; MNI: ±24, −14, −20), body (middle, mHipp; MNI: ±26, −26, −12), and tail (posterior, pHipp; MNI: ±26, −34, −4). The exact location of these nodes was based on previous human evidence with different anatomical and functional contours of the medial temporal lobe and functional consistency along the long axis of the hippocampus (Greicius et al., 2003). The MNI coordinates from the anterior, middle, and posterior nodes of interest are listed in Table 1. We selected these six regions in the left and right hippocampus as six ROIs to calculate the FC correlation coefficients across the whole brain. We extracted the BOLD time series and the seed regions from each subject, and then calculated Pearson correlation coefficients between the time series in each voxel and the time series of each voxel in the whole brain. The FC values for each voxel in the whole brain represent the FC value of the voxel with the seed region of interest, and the correlation coefficients (r values) were converted to z values for statistical analysis using Fisher z-transformation (Liu et al., 2017).

### Statistical Analysis

IBM SPSS 23.0 statistical software was used for the statistical analysis. The Kolmogorov–Smirnov test was performed to determine whether the data conformed to a normal distribution. To compare the differences in clinical evaluation between the OSA and GS groups, two-sample *t*-tests were used to analyse normally distributed data and Mann–Whitney *U*-tests were used to analyse non-normally distributed data. For the analysis of ROI FC, the single sample *t*-test was first used to explore the spatial distribution pattern of FC in each hippocampal subregion and the whole brain in the two groups. For the analysis of the resting-state FC (rs-FC) of each ROI, two independent samples *t*-tests were applied to analyze intergroup differences with age, BMI, and years of education as covariates. Multiple comparisons were corrected using Gaussian Random-Field method (voxel level, *p* < 0.005, cluster level, *p* < 0.05). Based on the linear correlation analysis, the rs-FC value of each ROI was associated with clinical variables, and *p* < 0.05 was considered to be statistical significance.

## Results

### Differences in Demographic and Clinical Characteristics

There were no significant differences in age or years of education between the OSA group and the GS group. However, significant differences in BMI, AHI, lowest blood oxygen saturation, average blood oxygen saturation, sleep time, sleep efficiency, N1, N2, REM, microarousal index, arterial oxygen saturation (SaO_2_) of <90%, ESS score, MoCA score, and other variables (see Table 1) were observed between the two groups.

### Resting-State Functional Connectivity Patterns Based on Seed Regions of Interest

**FIGURE 2:**
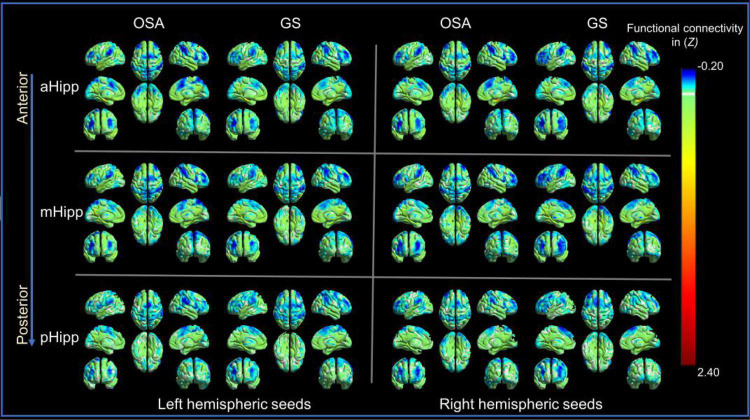
The distribution patterns of resting-state functional connectivity (FC) in different hippocampal subdivisions in the obstructive sleep apnoea (OSA) group and good sleepers (GS) group were highly similar. Panel (A) is for those in the OSA group; Panel (B) is for those in the GSs group.

The OSA patients and GSs showed similar resting-state functional connectivity patterns for the different hippocampal subdivisions, as shown in Figure 2.

### Differences in Functional Connectivity Between Hippocampal Subdivisions in Patients With Obstructive Sleep Apnoea

**FIGURE 3:**
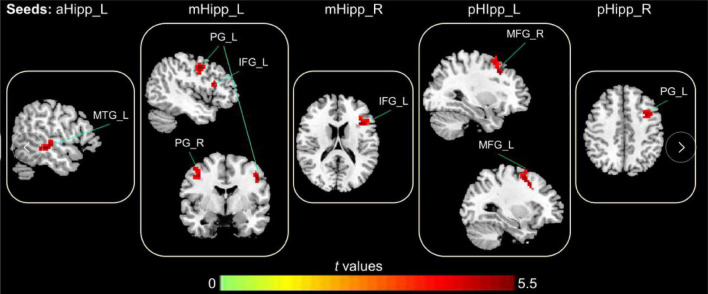
The abnormal FC in the hippocampal subdivision of OSA, the increased FC in OSA group compared to GSs group (Warm color); Gaussian Random-Field corrected; voxel level: *p* < 0.005; cluster volume > 10 voxels.

In the FC analysis of the hippocampal subdivision network, compared to GS group, the FC of the OSA group was significantly increased between the left anterior hippocampus and left middle temporal gyrus; between the left middle hippocampus and the left inferior frontal gyrus, right precentral gyrus, and left precentral gyrus; and between the left posterior hippocampus and both the right middle frontal gyrus and left middle frontal gyrus. Similarly, the FC between the right middle hippocampus and left inferior frontal gyrus was increased significantly, as was the FC between the right middle hippocampus and left middle frontal gyrus (Table 2 and Figure 3).

**TABLE 2:** Differences in functional connectivity (FC) of hippocampal subregions between OSA and GSs.

Hippocampal subregions	Brain regions	Voxel number	Peak MNI coordinates			*t*-value
			X	Y	Z	
aHipp-L	MTG-L	42	−54	−34	−6	4.9563
mHipp-L	IFG-L	43	−45	18	18	4.509
	PG-R	44	42	−3	42	4.5147
	PG-L	76	−48	−3	39	4.9761
pHipp-L	MFG-R	49	30	18	42	4.9982
	MFG-L	78	−30	9	45	5.0197
mHipp-R	IFG-L	128	−39	18	18	5.5911
pHipp-R	PG-L	36	−33	6	45	4.8155

*aHipp, anterior hippocampus; mHipp, middle hippocampus; pHipp, posterior hippocampus; L, left; R, right; MTG, Middle temporal gyrus; IFG, Inferior frontal gyrus; PG, Precentral gyrus; MFG, Middle frontal gyrus.*

### Correlations Between Changes in Functional Connectivity Between Hippocampal Subregions and Clinical Findings in Patients With Obstructive Sleep Apnoea

**FIGURE 4:**
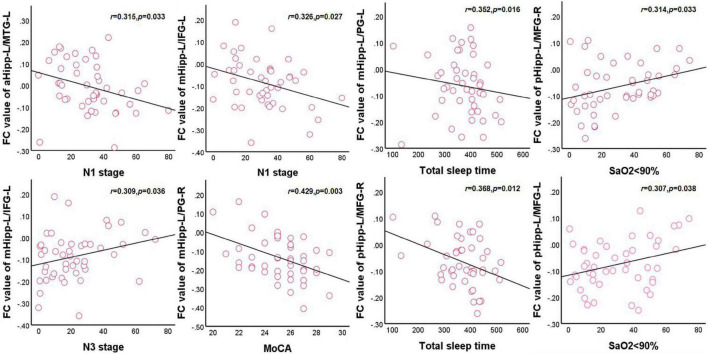
Correlations between hippocampal FC and sleep respiratory parameters and cognitive assessment scale in OSA. aHipp-L/MTG-L, left anterior hippocampus and left middle temporal gyrus; mHipp-L/IFG-L, left middle hippocampus and left inferior frontal gyrus; mHipp-L/PG-R, left middle hippocampus and right precentral gyrus; pHipp-L/MFG-R, left posterior hippocampus and right middle frontal gyrus; pHipp-L/MFG-L, left posterior hippocampus and left middle frontal gyrus.

In the OSA group, after adjusting for age, education level, and BMI, the FC values between the left anterior hippocampus and left middle temporal gyrus were negatively correlated with N1 (*r* = 0.315, *p* = 0.033) and positively correlated with orientation (*r* = 0.343, *p* = 0.019). The FC value between the left middle hippocampus and left inferior frontal gyrus was negatively correlated with N1 (*r* = 0.326, *p* = 0.027) and positively correlated with N3 (*r* = 0.309, *p* = 0.036). The FC value between the left middle hippocampus and right precentral gyrus was negatively correlated with the MoCA scores (*r* = 0.429, *p* = 0.003), orientation (*r* = 0.394, *p* = 0.007), and total sleep time (*r* = 0.352, *p* = 0.016). The FC value between the left posterior hippocampus and right middle frontal gyrus was negatively correlated with total sleep time (*r* = 0.368, *p* = 0.012) and attention (*r* = 0.344, *p* = 0.019) and positively correlated with an SaO_2_ of <90% (*r* = 0.314, *p* = 0.033). The FC value between the left posterior hippocampus and left middle frontal gyrus was positively correlated with an SaO_2_ of <90% (*r* = 0.307, *p* = 0.038). Finally, the FC value between the right middle hippocampus and left inferior frontal gyrus was positively correlated with naming (*r* = 0.309, *p* = 0.036; Figure 4).

## Discussion

This study aimed to investigate the changes in FC between hippocampal subdivisions and their relationship with neurocognitive function in patients with OSA. Our primary findings were as follows: First, FC between the hippocampal subregions and the middle temporal gyrus, inferior frontal gyrus, anterior central gyrus, and middle frontal gyrus, which involve the sensorimotor network, frontal parietal network, and semantic/default mode network, was enhanced in the OSA group relative to the GS group. Secondly, these abnormal FC patterns were significantly correlated with the MoCA score, sleep rhythm, and SaO_2_ of <90%. These result suggest a pattern separation of FC between the hippocampus subregions and the cerebral cortex. These changes are associated with intermittent hypoxia and abnormal sleep rhythms associated with OSA and provide additional information regarding the neural mechanisms underlying cognitive impairment. To the best of our knowledge, this study was the first to use resting-state fMRI to explore the FC patterns and changes between six subregions of the anterior and posterior axes of the hippocampus and the whole brain in male patients with OSA.

The FC between multiple subdivisions of the hippocampus and the cortex in the OSA group was significantly enhanced compared with that of the GS group in this study. It is well established that the dorsal and ventral sides of the hippocampus are divided into different neuroanatomical features, and the pattern of differences in connections between the anterior and posterior hippocampal axes and the cerebral cortex observed here is consistent with those found in animal models (Fanselow and Dong, 2010). Further, Dong et al. (2009) identified the genetic markers that revealed the existence of different spatial expression domains along the CA1 axis in the hippocampus and reported that the spatial domain of CA1 field was independently connected genetically, forming different functional networks related to emotion. Neuropsychological and functional neuroimaging studies in humans provide evidence for the separation of posterior and anterior hippocampal functions (Voets et al., 2014). Spatial navigation and memory behavior were found to be correlated with the dorsal hippocampus, while the stress response and emotional behavior were correlated with the ventral hippocampus (Kuhn and Gallinat, 2014). The left and right sides of the hippocampus contained different numbers of functional clusters, which also provided evidence for the functional differences between the two sides of the hippocampus (Robinson et al., 2015). Graph theory analysis revealed the continuous functional gradient of the anterior and posterior hippocampal network organization (Bullmore and Sporns, 2012). The node centrality of the posterior hippocampal subregion was higher than that of the anterior and middle hippocampal subregions, which was related to the fact that the posterior hippocampal subregion was more involved in medial temporal lobe network information integration (Strange et al., 2014). Macey et al. (2018b) showed that in OSA patients, the volume of the bilateral hippocampus CA1 area increased, the volume of inferior pate and uncinate gyrus increased, and the volume of the right hippocampus CA3 area decreased. This difference in hippocampal subregion volume was related to inflammation and varying degrees of local nerve damage. Therefore, we speculated that FC differences in the bilateral hippocampal subregions were related to the anatomical and genetic characteristics of the hippocampus and indirect hypoxia caused hippocampal damage while changing the functional connection mode between the hippocampal subregion and the cerebral cortex.

In this study, the FC between the left anterior hippocampus and the left middle temporal gyrus was enhanced. Middle temporal gyrus is a node in the default mode network (DMN), which is involved in the regulation of negative self-referential thinking in major depression (Solomonov et al., 2020). The anterior hippocampal subregion plays a leading role in processing and social emotional processing (Cohn et al., 2015; Adnan et al., 2016). Resting-state MRI studies of hippocampal subregions (Robinson et al., 2015) have demonstrated that healthy subjects have enhanced FC between the hippocampal head and multiple cerebral cortices, including the temporal cortex, precuneus, posterior cingulate gyrus, and parietal lobe. This enhanced FC plays a key role in emotional processing and neurocognition. Diffusion tensor imaging (DTI) studies have also observed specific connectivity features in areas of the anterior hippocampus involved in facial monitoring and emotion (Robinson et al., 2015). Consistent with previous studies, our results further confirm the enhanced FC between the anterior hippocampus and the cerebral cortex. In addition, we found increased FC between the loss of the anterior hippocampus and other cortical regions in patients with OSA compared to healthy subjects, and this relative reduction in FC may be the underlying cause of mood and cognitive impairment in OSA patients. We also found that FC enhancement in the left anterior hippocampus and left middle temporal gyrus was negatively correlated with N1, and the potential causal relationship remains unclear and needs further exploration.

The anterior central gyrus belongs to the sensorimotor network and is the location of the primary motor cortex (Zhou et al., 2020c). Structural and functional impairments in the anterior central gyrus, such as thinning of the motor network cortex in areas associated with upper airway motor control, have been reported in patients with OSA (Macey et al., 2018a). An fMRI study showed that reduced rs-FC in the anterior central gyrus was significantly associated with motor dysfunction in patients with OSA (Zhang et al., 2013). Our study suggests that the enhancement of FC in the central, posterior, and anterior central gyri may be a compensation mechanism for systemic muscle dysregulation, and that relaxation of the genioglossus and the upper airway pharyngeal dilator is associated with OSA severity. In addition, we found that FC enhancement in the left middle hippocampus and right anterior central gyrus was inversely correlated with the MoCA score, which may partly explain the information processing speed and executive dysfunction caused by impaired anterior central gyrus.

In addition, we found enhanced FC between the middle and posterior hippocampus and the middle frontal gyrus. The middle frontal gyrus belongs to the frontal-parietal control network, which is related to visuospatial planning, memory, attention, and cognitive control (Spreng et al., 2010). Zhang et al. (2013) reported that the structural and functional impairment of the frontal parietal network in patients with OSA was caused by hypometabolism of the prefrontal region and cortical atrophy. Zhou et al. (2020b) showed that the ReHO in the middle frontal gyrus of patients with moderate to severe OSA decreased, suggesting that intermittent hypoxemia at night may lead to impaired prefrontal lobe function, thus leading to cognitive impairment, which is thought to be due to a rearrangement of coherence and connectivity throughout the brain. Notably, we have observed the same results previously (Peng et al., 2014). Similar to previous results, we found that FC enhancement between the middle hippocampus, posterior hippocampus, and middle frontal gyrus was positively correlated with an SaO_2_ of <90%. Therefore, we speculate that this FC enhancement may serve as a potential compensation mechanism for the frontal parietal network affected by intermittent hypoxia injury.

The inferior frontal gyrus belongs to the semantic control network and is associated with controlled semantic retrieval (Thompson et al., 2015). Other brain regions associated with semantic control include the middle temporal gyrus (co-activated in active search tasks). Becker et al. (2020) showed that creative cognition may be the result of the joint activation of multiple brain regions, including the inferior frontal gyrus and middle temporal gyrus. Bai et al. (2021) indicated that ReHo in the left middle frontal gyrus and inferior frontal gyrus was significantly reduced in children with OSA, and the frontal lobe dysfunction was related to the underlying mechanism of internal brain activity. Our previous study (Yu et al., 2019) demonstrated that the FC between the amygdala and the left inferior frontal gyrus is enhanced in patients with OSA and is a compensatory mechanism for cognitive impairment. Interestingly, we found that the FC between the middle hippocampus and inferior frontal gyrus was positively correlated with naming and naming tasks activated brain regions of the semantic control network (Nettekoven et al., 2018). Therefore, we hypothesized that the FC enhancement between the hippocampal subregion, inferior frontal gyrus, and middle temporal gyrus may be a common compensation mechanism for the impaired semantic control network. We also found that the FC between the middle hippocampus and inferior frontal gyrus was negatively correlated with N1 and positively correlated with N3, which may be related to the circadian rhythm susceptibility and increased sleep disruption to neurodegeneration (Mattis and Sehgal, 2016).

### Limitations

This study has some limitations. First, the sample size was small, which may restrict the representativeness of the results. Second, only patients with severe OSA were included, and those with mild and moderate OSA were excluded. Furthermore, this study did not include female patients, and the sex differences in the hippocampus may limit the generalizability of the results to the whole population. Future studies should use a larger sample size of patients with OSA and include female patients.

## Conclusion

We observed abnormal functional connections between the hippocampal subregions and the whole brain in male patients with severe OSA, and these abnormal functional connections mainly involved the sensorimotor network, semantic control network, and frontoparietal network, which are related to memory and cognition. These findings may be beneficial for investigating the potential neural mechanisms underlying cognitive impairment in patients with OSA.

## Data Availability Statement

The raw data supporting the conclusions of this article will be made available by the authors, without undue reservation.

## Ethics Statement

The studies involving human participants were reviewed and approved by the Ethics Committee of The First Affiliated Hospital of Nanchang University and therefore been performed in accordance with the ethical standards outlined in the 1964 Declaration of Helsinki and its later amendments. The patients/participants provided their written informed consent to participate in this study. Written informed consent was obtained from the individual(s) for the publication of any potentially identifiable images or data included in this article.

## Author Contributions

XL and LC wrote, reviewed, and revised the manuscript. DP guided and designed the MRI experiment. LC analyzed the resting-state fMRI data and results and wrote the manuscript. XL and WD analyzed and discussed the ideas of the manuscript. HL, LK, YS, PL, WX, and YZ collected resting-state fMRI data and applied for the ethics approval. All authors contributed to the article and approved the submitted version.

## Conflict of Interest

The authors declare that the research was conducted in the absence of any commercial or financial relationships that could be construed as a potential conflict of interest.

## Publisher’s Note

All claims expressed in this article are solely those of the authors and do not necessarily represent those of their affiliated organizations, or those of the publisher, the editors and the reviewers. Any product that may be evaluated in this article, or claim that may be made by its manufacturer, is not guaranteed or endorsed by the publisher.

## Footnotes

1
www.MRIcro.com

2
https://www.fil.ion.ucl.ac.uk/spm/
